# A Patient-Independent Significance Test by Means of False-Positive Rates in Selected Correlation Analysis of Brain Multimodal Monitoring Data

**DOI:** 10.1155/2018/6821893

**Published:** 2018-08-08

**Authors:** Rupert Faltermeier, Martin A. Proescholdt, Stefan Wolf, Sylvia Bele, Alexander Brawanski

**Affiliations:** ^1^Department of Neurosurgery, University Hospital Regensburg, Regensburg, Germany; ^2^Department of Neurosurgery, University Hospital Charite, Berlin, Germany

## Abstract

Recently, we introduced a mathematical toolkit called selected correlation analysis (sca) that reliably detects negative and positive correlations between arterial blood pressure (ABP) and intracranial pressure (ICP) data, recorded during multimodal monitoring, in a time-resolved way. As has been shown with the aid of a mathematical model of cerebral perfusion, such correlations reflect impaired autoregulation and reduced intracranial compliance in patients with critical neurological diseases. Sca calculates a Fourier transform-based index called selected correlation (sc) that reflects the strength of correlation between the input data and simultaneously an index called mean Hilbert phase difference (mhpd) that reflects the phasing between the data. To reliably detect pathophysiological conditions during multimodal monitoring, some thresholds for the abovementioned indexes sc and mhpd have to be established that assign predefined significance levels to that thresholds. In this paper, we will present a method that determines the rate of false positives for fixed pairs of thresholds (lsc, lmhpd). We calculate these error rates as a function of the predefined thresholds for each individual out of a patient cohort of 52 patients in a retrospective way. Based on the deviation of the individual error rates, we subsequently determine a globally valid upper limit of the error rate by calculating the predictive interval. From this predictive interval, we deduce a globally valid significance level for appropriate pairs of thresholds that allows the application of sca to every future patient in a prospective, bedside fashion.

## 1. Introduction

In critical neurological pathologies such as subarachnoid hemorrhage (SAH) or traumatic brain injury (TBI), two major mechanisms of neuronal damage have been identified [1–3]. The primary injury consists of direct tissue damage due to contusion, laceration, or intracranial hemorrhage, which can only be influenced therapeutically to a limited degree. In contrast, secondary injury is caused by a self-propagating biochemical cascade leading to neuronal dysfunction and death over hours and weeks after the initial insult, which could be a potential treatment target. However, despite promising results of translational research in this field, the clinical studies applying neuroprotective compounds have been uniformly disappointing [4]. Due to the lack of causative treatment, the primary focus of neurointensive care therefore provides the optimal physiological environment in order to minimize secondary injury and foster early regenerative processes [5]. To achieve this goal, it is mandatory to detect pathophysiological conditions such as impaired cerebral autoregulation and reduced intracranial compliance prior to irreversible neuronal damage [6, 7]. Consequently, multimodal brain monitoring has been established to obtain a robust biophysical signature and to tailor an individualized therapy for each patient [8, 9].

In an earlier study, we could demonstrate with the aid of a mathematical model of cerebral perfusion and oxygen supply that severely reduced cerebral compliance in combination with a defective autoregulation leads to a positive correlation between ABP and ICP data, whereas a severely reduced cerebral compliance in combination with an intact cerebral autoregulation leads to a negative correlation between the abovementioned signals [10, 11]. Therefore, we have developed a mathematical toolkit called selected correlation analysis (sca) that reliably detects positive and negative correlations in ABP and ICP data recorded during multimodal monitoring at an intensive care unit.

This method calculates two indices, the selected correlation (sc) and the mean Hilbert phase difference (mhpd) of two isochronous data windows of ABP and ICP data, whereby the sc value serves as a measure for the strength of correlation between the data windows and mhpd reflects the phasing between the data windows. The medical relevance of positive correlations detected by calculation of sc and mhpd values was demonstrated in previous studies including a comparison with the well-established PRx calculations as an index for autoregulation failure [12]. The goal of this work is to assign significance levels to specified pairs of thresholds (lsc, lmhpd) to reliably detect the abovementioned pathophysiological conditions. Furthermore, to make this computerized analysis method available as a point of care tool to support goal-directed clinical decision making, we attempted to establish patient-independent significance levels for these pairs of thresholds which would allow to apply sca prospectively to every future patient in a prospective, bedside fashion.

## 2. Methods

### 2.1. Patient Population

To determine the significance of different threshold settings (lsc, lmhpd) and test the resulting error rates for normal distribution, we analyzed continuous measurements of ABP and ICP data of a patient cohort of 52 patients (32 female; 20 male) with a mean age of 50.4 years. The patients received multimodal brain monitoring either for the treatment of subarachnoid hemorrhage (*n*=43; 82.7%) or traumatic brain injury (*n*=9; 17.3%). A detailed description of the baseline characteristics is provided in Table 1. The study was performed in accordance to the Declaration of Helsinki and was approved by the local ethics review boards. The patients were treated either at the University Regensburg Medical Center (*n*=25; 48.1%) or at the University Hospital Charite, Berlin (*n*=27; 51.9%). The baseline parameters between the two patient subcohorts were balanced except for the diagnosis, which showed significantly more patients with SAH in the Berlin subcohort (*p*=0.001). Informed consent was obtained from the patients or their relatives; the data were stored and analyzed after anonymization according to the study protocol. Intracranial pressure (ICP) monitoring was carried out either via an external ventricular drain (EVD) or a parenchymal ICP probe (Raumedic, Helmbrechts, Germany). Follow-up was completed up to March 2017, the mean follow-up time was 53.8 months, and no patient was lost for follow-up. The neurological outcome was measured by the Glasgow Outcome Scale at the last follow-up, and the median score was 3 (range: 1–5, Table 1).

### 2.2. Mathematical Framework of Selected Correlation Analysis

**Figure 1:**
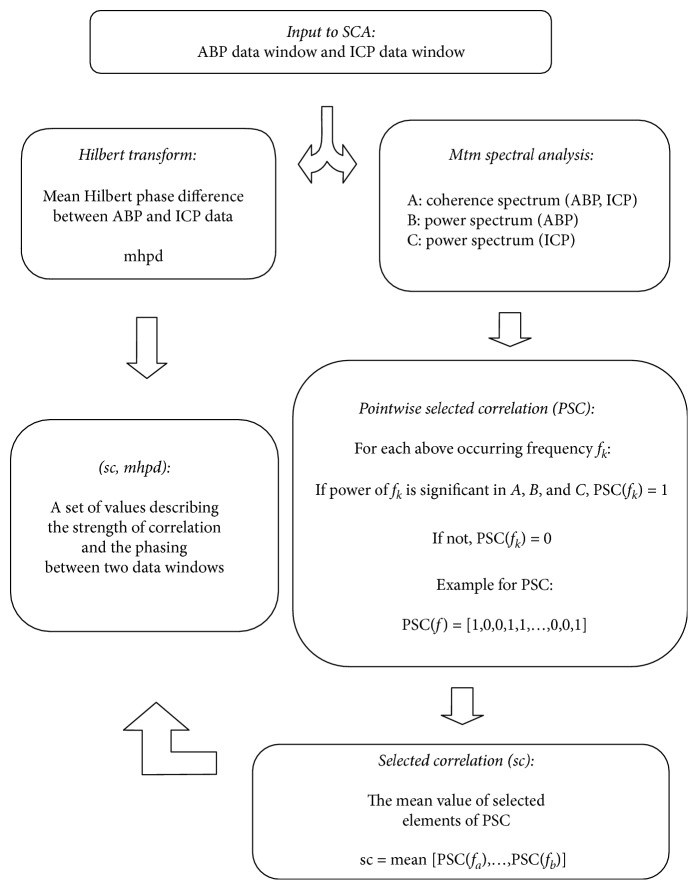
Selected correlation analysis (sca) illustrated as flowchart depicting the different elements of the method.

Selected correlation analysis (sca) is a method to detect correlations between two data windows of fixed length. The different elements of this analysis method are illustrated in Figure 1. Thereby, information about correlation is gained by fast Fourier transform of the data and subsequent analysis in frequency space. This approach permits the detection of correlation in a specific frequency band, allowing a differentiation between the correlation of fast or slow components of the signals. Additionally, the whole spectral information about correlation is condensed to a simple value, called sc, which serves as a measure for the degree of correlation between two data windows in a specific frequency range [13, 14].

Let *X*:={*x*_1_,…,  *x*_*N*_} be a time series of ABP or ICP data of length *N*. A fixed segment or window *X*^*s*, *k*^(*t*) of such a time series can be represented as a function in time. An individual window is defined by its starting point *k* and its length *s*:

(1) $$\begin{array}{c} X^{s,\;k}\left(t\right)≔\left\{x_{k+t-1}\in X\;|\;1\leq t\leq s;\;k+s-1\leq N;\;s\equiv 2^{u\in \mathbb{N}}\right\}. \end{array}$$

In the following, we will use such windows of ABP and ICP data as input for multitaper power spectrum (mtms) analysis and multitaper coherence spectrum (mtmc) analysis [15]. This spectral analysis will transform the discrete time domain of the windows, indexed by *t*, into the discrete frequency domain of the spectra, indexed by *f* with range 1 ≤ *f* ≤ *s*/2:

(2) $$\begin{array}{c} S^{s}\left(k,\;f\right)≔\text{mtms }\mathrm{of}\;X^{s,k}\left(t\right),\\ C^{s}\left(k,\;l,\;f\right)≔\text{tmc }\mathrm{of}\;X^{s,k}\left(t\right)\;\mathrm{and}\;Y^{s,l}\left(t\right). \end{array}$$

The multitaper method comes along with a built-in statistical test for the significance of each single frequency *f*. Using this significance test for each individual frequency, we define the so-called pointwise selected correlation (PSC):

(3) $$\begin{array}{c} {\text{PSC}}^{s}\left(k,\;l\right)≔\left({\text{psc}}_{1},\ldots ,\;{\text{psc}}_{s/2}\right)\text{ with}\\ {\text{psc}}_{f}≔\left\{\begin{array}{cc} 1, & \text{if }S^{s}\left(k,\;f\right)\;\wedge \;S^{s}\left(l,\;f\right)\;\wedge \;C^{s}\left(k,\;l,\;f\right)\text{ significant},\\ 0, & \text{otherwise}. \end{array}\right. \end{array}$$

The above-defined PSC tuple contains information whether a frequency is significant in all three spectra, or not. Being significant in both power spectra assures that the specific frequency contributes essentially to the original signals. If this frequency is additionally significant in the coherence spectrum, a strong correlation between the input signals for this frequency is implied. Using *N* successive pairs of isochronous windows as input for the above-described PSC calculations produces a time-resolved sequence of PSC tuples. From this sequence, we can deduce a measure for the average activity of the frequencies by calculating the mean pointwise selected correlation (MPSC):

(4) $$\begin{array}{c} {\text{MPSC}}^{s}\left(f\right)≔\left(\frac{1}{N}\right)\;*\;\overset{j=N}{\underset{j=1}{\sum }}{\text{PSC}}_{f}^{s}\left(j,\;j\right). \end{array}$$

The MPSC tuple is used to potentially identify frequency intervals, which basically carry information about correlations between the input data. Having found such a frequency interval *U*=(*m*,…,  *n*), the next step is to determine time sequences in the data sets, where strong correlations occur with respect to *U*. A simple measure for the strength of correlation of a pair of windows with respect to *U* is gained by summing up all elements of the appropriate PSC tuple belonging to *U* and dividing this sum by the length of *U*. This measure is called selected correlation (sc):

(5) $$\begin{array}{c} {\text{sc}}^{s,m,n}\left(k,\;l\right)≔\frac{1}{n-m+1}\overset{f=n}{\underset{f=m}{\sum }}{\text{PSC}}_{f}^{s}\left(k,\;l\right),\\ \text{with }1\leq m<n\leq \frac{s}{2}. \end{array}$$

Predefining a threshold lsc, a pair of windows will be called selected correlated if sc > lsc. Using isochronous windows as input while shifting the starting points $\tilde{t}$ along the time axis produces time-resolved information about the degree of correlation:

(6) $$\begin{array}{c} {\text{sc}}^{s,m,n}\left(\tilde{t}\right)≔\;\frac{1}{n-m+1}\overset{f=n}{\underset{f=m}{\sum }}{\text{PSC}}_{f}^{s}\left(\tilde{t},\;\tilde{t}\right). \end{array}$$

#### 2.2.1. Hilbert Phase Differences

With the abovementioned sc index, we can identify windows exhibiting a strong correlation between the input data. In order to assign the model predicted pathophysiological conditions, identified by positive and negative correlations, we also have to determine the phasing of the input data. The phasing of the data can be determined by using the so-called Hilbert transform, a mathematical approach to transform a real-valued function *s*(*t*) into the complex plain:

(7) $$\begin{array}{c} s_{\text{analytic}}\left(t\right)≔s\left(t\right)+i\text{⁡}*\text{⁡}\overset{˜}{s}\left(t\right)=A\left(t\right)\text{⁡}*\text{⁡}e^{i*\varphi \left(t\right)},\\ \text{with}\overset{˜}{s}\left(t\right)≔\pi^{-1}P\cdot V\cdot \int_{-\infty }^{\infty }\frac{s\left(\tau \right)}{t-\tau }\text{⁡}d\tau . \end{array}$$

By calculating the Hilbert transformation of two windows *X*^*s*, *k*^(*t*),  *Y*^*s*, *l*^(*t*), we are able to determine the associated phases *φ*_*X*_(*t*),  *φ*_*Y*_(*t*) of the data and the Hilbert phase difference hpd(*t*):

(8) $$\begin{array}{c} \text{hpd}\left(t\right)≔\varphi_{X}\left(t\right)-\varphi_{Y}\left(t\right)=\arctan\left(\frac{{\tilde{X}}^{s,\;k}\left(t\right)Y^{s,\;l}\left(t\right)-X^{s,\;k}\left(t\right){\tilde{Y}}^{s,\;l}\left(t\right)}{X^{s,\;k}\left(t\right)Y^{s,\;l}\left(t\right)-{\tilde{X}}^{s,\;k}\left(t\right){\tilde{Y}}^{s,\;l}\left(t\right)}\right). \end{array}$$

As a simple measure for the phasing of two windows, we will use the mean value mhpd of hpd(*t*):

(9) $$\begin{array}{c} {\text{mhpd}}^{s}\left(k,\;l\right)≔\frac{1}{s}*\underset{t}{\sum }\text{hpd}\left(t\right). \end{array}$$

Analogous to the sc value, a pair of windows is called positively correlated (scp) if sc > lsc and mhpd < lmhpd_pos_. If sc > lsc and mhpd > lmhpd_neg_, the data windows will be called negatively correlated (scn).

### 2.3. Construction of Patient-Independent Significance Levels

#### 2.3.1. Statistical Test

With the aid of sc and mhpd values, we are able to identify positively and negatively correlated sections of the input data, but up to now, we do not know the specificity of such correlations. Therefore, we will establish a statistical test, which allows us to relate a significance to individual pairs of the thresholds (lsc, lmhpd_pos_) and (lsc, lmhpd_neg_) in a patient-independent fashion, so that the resulting threshold pairs can be used for prospective studies. This statistical test uses the fact that the mathematical model predicts isochronous correlations between ABP and ICP. Consequently, two segments of ABP and ICP with starting points far apart from each other should not correlate. If, for example, we use data windows containing one hour of data and a starting point of the ICP data, that is, five hours later than the starting point of the ABP data, then there should be no casual link between these data windows and therefore no correlation. But, due to measurement noise, a few of the frequencies included in the sc analysis may exhibit a significant correlation.

Using this observation, we can count how often such separated windows produce, for example, values (sc, mhpd_pos_) higher than a predefined pair of threshold levels (lsc, lmhpd_pos_). The resulting error rate for this fixed pair of thresholds then determines the significance for this specific pair of thresholds. Clearly, this only applies to cases where the offset between the input data windows is big enough to avoid autocorrelation effects. Such effects can be estimated using the so-called mean windowed autocorrelation (mwa):

(10) $$\begin{array}{c} {\text{mwa}}^{s,R,m,n}\left(o\right)≔\left(\frac{1}{R}\right)\overset{i=R}{\underset{i=1}{\sum }}{\text{sc}}^{s,m,n}\left(k_{i},\;k_{i}+o\right),\\ \text{with }k_{i}\text{ random}. \end{array}$$

For offset *o* big enough to avoid autocorrelation, the values of mwa should become small and stable. With the knowledge of an appropriate offset *o*, we are able to calculate the error indices *ei*^*s*,*m*,*n*^(*k*,  *l*,  lsc,  lmhpd_pos/neg_), for a predefined pair of thresholds (lsc, lmhpd_pos/neg_) and a fixed pair of input windows with starting points *k* and *l* satisfying *l* > *k*+*o*:

(11) $$\begin{array}{c} ei^{s,m,n}\left(k,\;l,\;\mathrm{lsc},\;{\text{lmhpd}}_{\text{pos}}\right)≔\left\{\begin{array}{c} 1,\;\text{if }{\text{sc}}^{s,m,n}\left(k,\;l\right)>\mathrm{lsc}\;\wedge \;\mathrm{mhpd}\left(k,\;l\right)<{\text{lmhpd}}_{\text{pos}},\\ 0,\;\text{otherwise}, \end{array}\right.\\ ei^{s,m,n}\left(k,\;l,\;\mathrm{lsc},\;{\text{lmhpd}}_{\text{neg}}\right)≔\left\{\begin{array}{c} 1,\;\text{if }{\text{sc}}^{s,m,n}\left(k,\;l\right)>\text{lsc }\wedge \;\mathrm{mhpd}\left(k,\;l\right)>{\text{lmhpd}}_{\text{neg}},\\ 0,\;\text{otherwise}. \end{array}\right. \end{array}$$

Repeating this *R* times for different starting points leads to the error rate asc:

(12) $$\begin{array}{c} {\text{asc}}^{s,R,m,n,o}\left(\mathrm{lsc},\;{\text{lmhpd}}_{\text{pos}/\text{neg}}\right)≔\left(\frac{1}{R}\right)\overset{i=R}{\underset{i=1}{\sum }}ei^{s,m,n}\left(k_{i},\;l_{i},\;\mathrm{lsc},\;{\text{lmhpd}}_{\text{pos}/\text{neg}}\right),\\ \text{with}k_{i}\text{ random}:l_{i}>k_{i}+o, \end{array}$$

which indicates the percentage of obviously uncorrelated input windows that induce (sc, mhpd_pos/neg_) values higher than some predefined thresholds (lsc, lmhpd_pos/neg_). In other words, the error rate asc represents the percentage of false positives. Therefore, for a particular pair of thresholds (lsc, lmhpd_pos/neg_), we are now able to assign a significance to the detection of windows labeled scp or labeled scn:

(13) $$\begin{array}{c} {\text{sig}}_{\text{scp}/\text{scn}}\left(\text{lsc},\;{\text{lmhpd}}_{\text{pos}/\text{neg}}\right)=\left(1-{\text{asc}}^{s,R,m,n,o}\left(\text{lsc},\;{\text{lmhpd}}_{\text{pos}/\text{neg}}\right)\right)\;*\;100. \end{array}$$

#### 2.3.2. Patient-Independent Statistics

Now, we can use the above-described method to calculate the significance for a specific pair of thresholds (lsc, lmhpd_pos/neg_) for a specific patient using the patient's ABP and ICP time series as input selecting an offset *o* big enough to avoid autocorrelation effects. However, if we apply the same approach to a different patient, the result for the identical thresholds may slightly vary, due to the individual conditions of measurement setup and noise components. In order to determine a significance level for a specific pair of thresholds that is universally valid, we use the following approach.

For a fixed pair of thresholds (lsc, lmhpd_pos/neg_), we first calculate the appropriate error rates asc(lsc,  lmhpd_pos/neg_) for each patient included in the study. Then, we check whether the distribution of the resulting error rate values is normal or not. In case of a normal distribution, we are able to deduce an upper limit lasc_scp/scn_^up^=*μ*+*z* · *σ* from the one-sided prediction interval [−*∞*,  *μ*+*z* · *σ*] for different probability levels *c*(*z*) ∈ [0,  1] defined by the abovementioned standard score *z* [16]. The upper limit of the one-sided prediction interval represents a value that assures that all future measurements will produce error rates lower than this value with a probability of *c*(*z*). Thus, the patient-independent significance for this specific pair of thresholds could be defined as follows:

(14) $$\begin{array}{c} {\text{sig}}_{\text{scp}/\text{scn}}^{\text{indep}}=c\left(z\right)\;*\;\left(1-{\text{lasc}}_{\text{scp/scn}}^{\text{up}}\right)\;*\;100. \end{array}$$

#### 2.3.3. Statistics of the Individual Error Rates

For each set of error rates asc(lsc,  lmhpd_pos/neg_), we computed mean, median, maximal/minimal values, standard deviation and error as well as variance, and 90–99% one-sided prediction intervals as population parameters. Subsequently, the error test results derived from different parameter settings of sca were analyzed for normal distribution using skewness–kurtosis testing (modified Jarque–Bera test). Differences in rates and proportions were analyzed by contingency tables and chi-square testing. Two-group comparisons were performed by computing Wilcoxon rank-sum tests.

## 3. Results

For the subsequent analysis, we used ABP and ICP data with sample frequency of 0.2 Hz and a window size *s* of 1024 points. The frequency band was set to *f* ≤ 0.00684 Hz, an offset *o* of 5000 points was used, and *R* was set to one million [14]. With this, we calculated the error rates asc for different pairs of thresholds (lsc, lmhpd_pos/neg_) consisting of the first 5 possible lsc values (0.000, 0.028, 0.056, 0.083, and 0.111) combined with lmhpd values from 50–80 degrees with a one-degree step size, for each patient separately.

**Figure 2:**
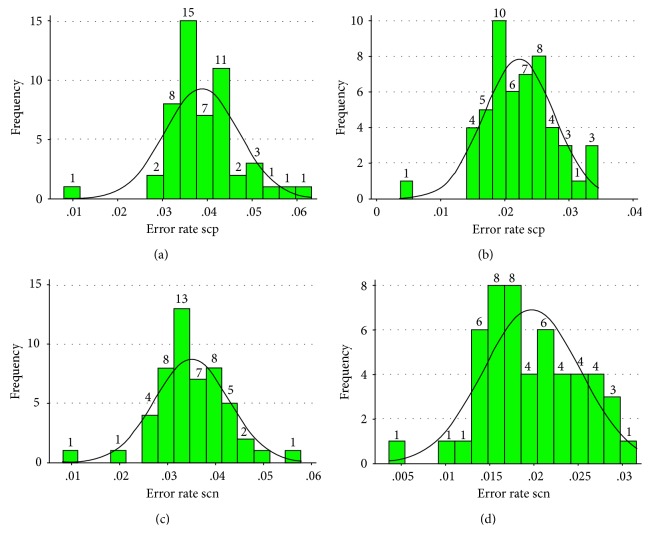
Frequency histograms illustrating the specific error rates for (a) scp lsc 0.056/lmhpd, (b) scp lsc 0.056/lmhpd 60, (c) scn lsc 0.056/lmhpd 110, and (d) scn lsc 0.056/lmhpd 120. The resulting error rates from all four parameter settings were defined to be normally distributed (modified Jarque–Bera test).

**Figure 3:**
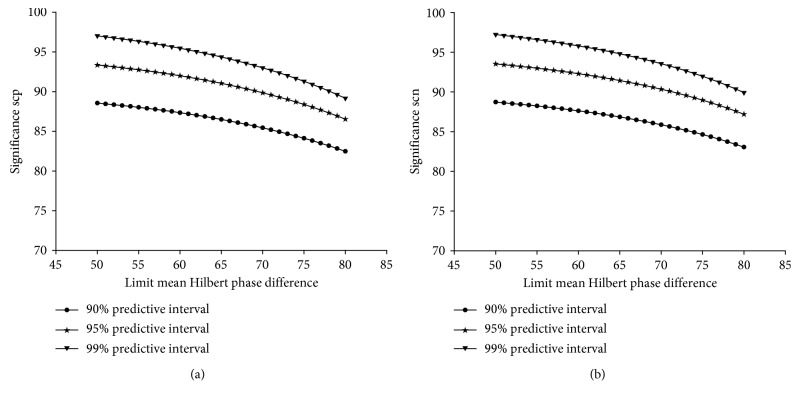
Relationship between patient-independent significance and mean Hilbert phase difference for scp (a) and scn (b).

The resulting error rate means for scp per parameter set ranged from 0.0033 to 0.205 and for scn from 0.0027 to 0.198. The scp-specific error rates were found to be normally distributed in a range of parameter settings from lsc 0.000/lmhpd 51 to lsc 0.111/lmhpd 50; in contrast, the scn-specific error rates were normally distributed from lsc 0.000/lmhpd 54 to lsc 0.083/lmhpd 66. Interestingly, the parameter settings, previously defined to be optimal for the clinical application of scp (lsc 0.056/lmhpd 70) [14], were found to be normally distributed for both scp- and scn-specific error test rates (Figure 2). To evaluate a threshold value, which would encompass any future patients for the error test result, we calculated the one-sided predictive intervals for 90–99% probability levels utilizing the error test results of our retrospective patient cohort [16]. The predictive intervals for both scp and scn analysis are listed in Table 2. Utilizing the upper limit of the 99% prediction interval would allow us to extrapolate that the error test result of any future patient will be within the determined interval with a probability of 99%. In addition, we calculated the resulting patient-independent significances according to the abovementioned formula. The results of this calculation are illustrated in Figure 3.

## 4. Discussion

The implementation of multimodal brain monitoring into neurointensive care management has two primary goals: (a) to detect reduced intracranial compliance due to brain edema, hydrocephalus, stroke, or intracranial hemorrhage [17] and (b) to uncover failure of the cerebral autoregulation [18, 19]. Since both aspects critically determine the therapeutic regimen, it would be ideal to have an integrative, computerized platform available, which unmasks these critical events in a timely fashion. Several indices focusing exclusively on autoregulation failure have been evaluated primarily to allow a more precise prognosis regarding mortality and functional outcome in patients with TBI and SAH [20]. Our approach, termed “selected correlation analysis” (sca) provides a mathematical tool set which allows the detection of both mechanisms, autoregulation failure and impaired intracranial compliance [13]. Regarding reduced intracranial compliance, we have validated our method utilizing a serial CT imaging approach [21]. In contrast, the sensitivity and specificity of autoregulation failure detection was substantiated by the comparison of our approach with the pressure reactivity index (PRx) as an established marker [12, 19]. One of the most significant drawbacks in time series analysis is type one error induced by autocorrelation effects, leading to potentially inadequate clinical treatment decisions [22, 23]. To ensure the exclusion of false-positive readings due to potential autocorrelation [24], we have implemented an error test into our method, which was hitherto calculated utilizing a retrospective patient cohort. However, for the bedside application of a sca monitor, it was mandatory to establish patient-independent significance levels for scp and scn. Statistically, this can be achieved by computing the prediction intervals of the 52 analyzed patients which would serve as a learning cohort. The upper patient-independent significance for the detection of scp deduced from the learning cohort does not profoundly differ from the values calculated for the sca parameter set optimization utilizing that cohort [14]. Additionally, the corresponding results for scn show very similar properties, and the applied statistical framework is capable of improving the resulting patient-independent significances with new patients added to the analysis and therefore enhancing the sample size. As a limitation of our study, the results of our error test calculation are based on a limited number of patients. It is conceivable that with increasing case numbers, the significances will be adjusted with the consequence of a higher sensitivity. In conclusion, our results provide a patient-independent pairs of threshold values for both scp and scn. Following the development of this patient-independent significance test for false-positive readings, we are now able to apply our method as a point of care system in a prospective fashion.

## Figures and Tables

**Table 1:** Baseline characteristics of the retrospective patient cohort treated for subarachnoid hemorrhage (SAH) or traumatic brain injury (TBI).

Parameter	Number (%)
*N*	52
Gender (f/m)	32/20 (61.5/38.5)
Age (mean)	50.4 (range: 16.4–72.4)
Diagnosis (SAH/TBI)	43/9 (82.7/17.3)
GCS at admission (median)	7 (range: 3–14)
GOS at last follow-up (median)	3 (range: 1–5)

*Note*. The retrospective patient cohort was analyzed for false-positive readings of the sca method. To illustrate the initial clinical condition and patient outcome, the Glasgow Coma Scale (GCS) rates at admission and the Glasgow Outcome Score (GOS) value at last follow-up are reported.

**Table 2:** One-sided prediction intervals with upper limits of error rates for 90%, 95%, and 99% probability levels.

Analysis type	Prediction interval			Patient-independent significance
Scp lsc 0.056/lsc 60 lsc 0.056/lmhpd 70	90% 0.0294 0.0506	95% 0.0316 0.0541	99% 0.0357 0.0606	95.41 93.07

Scn lsc 0.056/lmhpd 120 lsc 0.056/lmhpd 110	0.0264 0.0459	0.0285 0.0490	0.0324 0.0550	96.65 93.57

*Note*. The resulting patient-independent significance values for scp and scn are listed in the last column.

## Data Availability

Data are available on request due to privacy or other restrictions.
